# Frequency of disturbance alters diversity, function, and underlying assembly mechanisms of complex bacterial communities

**DOI:** 10.1038/s41522-019-0079-4

**Published:** 2019-02-11

**Authors:** Ezequiel Santillan, Hari Seshan, Florentin Constancias, Daniela I. Drautz-Moses, Stefan Wuertz

**Affiliations:** 1 Singapore Centre for Environmental Life Sciences Engineering, Nanyang Technological University, Singapore, 637551 Singapore; 2 Department of Civil and Environmental Engineering, University of California, Davis, CA 95616 USA; 3 School of Civil and Environmental Engineering, Nanyang Technological University, Singapore, 639798 Singapore; 4 Present Address: Brown and Caldwell, 9665 Chesapeake Drive, Suite 201, San Diego, CA 92123 USA

## Abstract

Disturbance is known to affect the ecosystem structure, but predicting its outcomes remains elusive. Similarly, community diversity is believed to relate to ecosystem functions, yet the underlying mechanisms are poorly understood. Here, we tested the effect of disturbance on the structure, assembly, and ecosystem function of complex microbial communities within an engineered system. We carried out a microcosm experiment where activated sludge bioreactors operated in daily cycles were subjected to eight different frequency levels of augmentation with a toxic pollutant, from never (undisturbed) to every day (press-disturbed), for 35 days. Microbial communities were assessed by combining distance-based methods, general linear multivariate models, α-diversity indices, and null model analyses on metagenomics and 16S rRNA gene amplicon data. A stronger temporal decrease in α-diversity at the extreme, undisturbed and press-disturbed, ends of the disturbance range led to a hump-backed pattern, with the highest diversity found at intermediate levels of disturbance. Undisturbed and press-disturbed levels displayed the highest community and functional similarity across replicates, suggesting deterministic processes were dominating. The opposite was observed amongst intermediately disturbed levels, indicating stronger stochastic assembly mechanisms. Trade-offs were observed in the ecosystem function between organic carbon removal and both nitrification and biomass productivity, as well as between diversity and these functions. Hence, not every ecosystem function was favoured by higher community diversity. Our results show that the assessment of changes in diversity, along with the underlying stochastic–deterministic assembly processes, is essential to understanding the impact of disturbance in complex microbial communities.

## Microbial ecology: Disturbance affects microbial diversity and ecosystem function

Complex microbial communities and ecosystems are highly sensitive to disturbance, which can affect community diversity and structure, but to date the impact of disturbance remains difficult to predict. Here, Stefan Wuertz and colleagues from the Nanyang Technological University in Singapore show how different disturbance frequencies affect microbial population dynamics. Analyses of microbial communities in sludge bioreactors exposed to a toxic pollutant at different rates revealed that populations at the extremes (not exposed and most exposed) showed the lowest α-diversity, whereas populations exposed at intermediate levels were most diverse. Notably, ecosystem function trade-offs were observed between organic carbon removal and nitrification and biomass productivity, with diversity also affecting these functions. These observations highlight the importance of evaluating diversity when determining the effects of disturbance on microbial communities.

## Introduction

Understanding what drives patterns of community succession and structure remains a central goal in ecology^1,2^ and microbial ecology,^3^ especially since community diversity and assembly are thought to regulate the ecosystem function.^4,5^ Assembly processes can be either stochastic, assuming that all species have equal fitness and that changes in structure arise from random events of ecological drift,^6^ or deterministic, when communities form as a result of niche diversity shaped by abiotic and biotic factors.^7^ Deterministic and stochastic assembly dynamics have been proposed to simultaneously act in driving assembly patterns observed in nature.^8–12^ This has stimulated scientific discourse including modelling of experimental data^13–16^ and both observational and manipulative experimentation in a variety of ecosystems, like deserts on a global scale,^17^ groundwater,^18^ subsurface environments,^2,19,20^ soil plant–fungi associations,^21^ rock pools,^22^ water ponds,^23^ and sludge bioreactors.^15,24,25^ These prior studies emphasized the need to understand what governs the relative balance between stochastic and deterministic processes and what conditions would lead to stochastic processes overwhelming deterministic processes, particularly under disturbance.^20^ To investigate their roles, well-replicated time series experiments are needed.^18,25^

Disturbance is defined in ecology as an event that physically inhibits, injures, or kills some individuals in a community, creating opportunities for other individuals to grow or reproduce.^26^ When disturbance is long-term or continuous, it is classified as press-disturbance.^27^ Disturbance is deemed the main factor influencing variations in species diversity^28^ and structuring of ecosystems,^27,29^ but a clear understanding of its outcomes is lacking.^30^ Particularly, the intermediate disturbance hypothesis (IDH)^31^ predicts that diversity should peak at intermediate levels of disturbance due to trade-offs between species’ ability to compete, colonize ecological niches, and tolerate disturbance. The IDH has been influential in ecological theory, as well as in management and conservation,^32^ but its predictions do not always hold true.^28,33^ For example, in soil and freshwater bacterial communities, different patterns of diversity were observed with increasing disturbance frequency with biomass destruction^34^ and removal^35^ as disturbance type, respectively. Meanwhile, the effect of varying frequencies of non-destructive disturbances on bacterial diversity remains unknown. Furthermore, the IDH predicts a pattern but it is not a coexistence mechanism as it was originally purported to be.^36^ Hence, its relevance is being debated^37,38^ with multiple interpretations and simplicity as the main points of critique. To date, the mechanisms behind the observed patterns of diversity under disturbance remain to be elucidated.^39,40^

The objective of this work was to test the effect of disturbance on the bacterial community structure, diversity, and ecosystem function of a complex bacterial system, with emphasis on the underlying assembly mechanisms. We employed sequencing batch bioreactors inoculated with activated sludge from an urban wastewater treatment plant, in a laboratory microcosm setup with eight different frequency levels of augmentation with toxic 3-chloroaniline (3-CA) as disturbance. Triplicate reactors received 3-CA either never (L0, undisturbed), every 7, 6, 5, 4, 3, and 2 days (L1–6, intermediately-disturbed), or every day (L7, press-disturbed) for 35 days. Chloroanilines are toxic and carcinogenic compounds and few bacteria encode the pathways to degrade 3-CA,^41^ which is also known to inhibit both organic carbon removal and nitrification in sludge reactors.^42^ Microcosm studies are useful models of natural systems,^43^ can be coupled with theory development to stimulate further research,^44^ and by permitting easier manipulation and replication can allow inference of causal relationships^45^ and statistically significant results.^46^

We analysed changes in the ecosystem function over time by measuring the removal of organic carbon, ammonia, and 3-CA, as well as biomass. Changes in community structure were examined at different levels of resolution using a combination of metagenomics sequencing and 16S rRNA gene fingerprinting techniques. Such changes were assessed by employing a combination of ordination tools, diversity indices, cluster analysis, univariate and multivariate statistical analyses. We also explored how diversity was related to function, focusing on trade-offs. Furthermore, the role of stochasticity in community assembly was investigated by employing null model techniques from ecology. We hypothesized that time would lead to a decrease in α-diversity at the extreme sides of the disturbance range due to deterministic adaptation to the environment, while less predictable conditions at intermediate disturbance levels would lead to higher α-diversity and stochastic assembly. Consequently, replicates at intermediately disturbed levels should display higher variability in terms of both community structure and function, compared with the ones at the extreme sides of the disturbance range where the opposite (that is, less variability) should occur.

## Results and discussion

### Overall community dynamics and differentiation of clusters

**Fig. 1:**
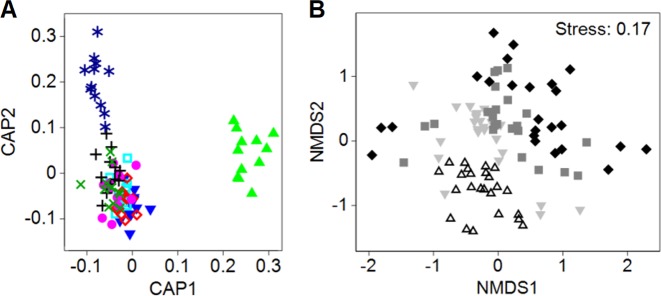
Microbial community dynamics across disturbance frequencies and time, as assessed by 16S rRNA gene terminal restriction fragment length polymorphism (T-RFLP) fingerprinting. **a** Canonical analysis of principal coordinates (CAP, constrained ordination) plot, with disturbance levels as differentiation criteria, shows cluster differentiation for L0 (CAP1 axis) and L7 (CAP2 axis) from intermediately disturbed levels (L1–6). Disturbance levels: L0 [light-green triangles], L1 [blue upside-down triangles], L2 [light-blue open squares], L3 [open red rhombuses], L4 [purple circles], L5 [black crosses], L6 [green x-symbols], and L7 [blue stars]. **b** Non-metric multidimensional scaling (NMDS, unconstrained ordination) shows temporal dispersion effect. Days: 14 [open triangles], 21 [light-grey upside-down triangles], 28 [dark-grey squares], and 35 [black rhombuses]

Bacterial community structure displayed temporal changes and varied between disturbance levels, as assessed by 16S rRNA gene terminal restriction fragment length polymorphism (T-RFLP) (Fig. 1). Constrained ordination showed a defined cluster separation with 0% misclassification error of the outermost levels L0 and L7 from the remaining intermediate levels L1–6 (Fig. 1a). Overall community structure differed over time with a dispersion effect after 14 days (Fig. 1b). Levels across disturbance and time factors showed significant differences (PERMANOVA *P* = 0.003, Supplementary Table 1), with a non-significant interaction effect (*P* = 0.15). Disturbance was the factor responsible for the observed clustering (Fig. 1a) and not heteroscedasticity (PERDIMSP *P* = 0.35).

### Ecosystem function dynamics and trade-offs

**Fig. 2:**
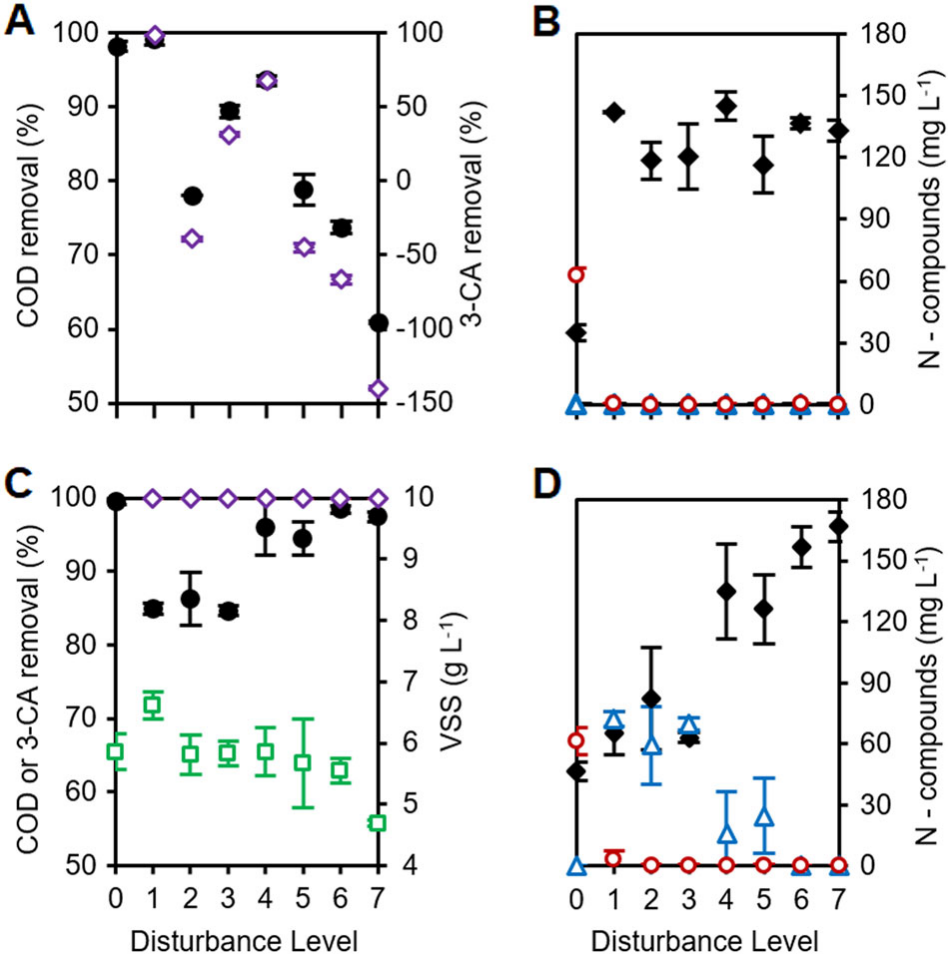
Process performance indicators across disturbance levels. Effects include temporal changes and trade-offs in community function. **a**, **c** Percentage of organic carbon as chemical oxygen demand (COD, black circles) and 3-CA (open purple rhombuses) removal for all levels (negative values represent accumulation). **c** Biomass as volatile suspended solids (VSS, open green squares). **b**, **d** Concentration of ammonium (black rhombuses), nitrite (open blue triangles), and nitrate (open red circles) as nitrogen for all levels. Data are from days 7 (**a**, **b**) and 35 (**c**, **d**) of the study (for all time points sampled, see Supplementary Figure 2). Mean ± s.d. (*n* = 3) are shown. Undisturbed L0 replicates had consistent organic carbon removal and complete nitrification, whereas press-disturbed L7 never showed nitrification and had the lowest final biomass. Intermediate levels L1–6 displayed changing functionality with higher s.d. values that increased over time

The undisturbed community (L0) was the only one with complete dissolved organic carbon (COD) removal and nitrate generation without nitrite residuals, while the press-disturbed community (L7) was the only one where nitrification products were never detected and also had the lowest biomass (Fig. 2). Initially, reactors at the disturbed levels showed an inability to remove all of the 3-CA (with the exception of L1). Such lack of 3-CA degradation was accompanied by a reduction in organic carbon removal in the first 3 weeks (Fig. 2a, Supplementary Figure 2a,c,e), and a complete inhibition of nitrification with subsequent accumulation of ammonium (Fig. 2b, Supplementary Figure 2b,d,f). Removal of 3-CA recovered and was above 95% for all disturbed levels after 28 days (Supplementary Figure 2g), but COD removal was still not 100% despite complete 3-CA removal towards the end of the experiment (Fig. 2c).

Nitrification was detected on day 21 for L1 and later for other disturbance levels, except for the press-disturbed L7. The dominant NO_X_ component was nitrite, but some nitrate was also produced (Fig. 2d, Supplementary Figure 2h–j). At the end of the study, there was a significant negative Spearman’s correlation between organic carbon removal and nitrification (Supplementary Figure 3a-b) in terms of nitrite (*ρ* = −0.901) and nitrate production (*ρ* = −0.697). Biomass values on day 35 differed significantly among levels with the highest value at L1 and the lowest at L7 (Fig. 2c). There was a significant positive correlation between biomass and nitrification in terms of nitrite (*ρ* = 0.466) and nitrate production (*ρ* = 0.656) (Supplementary Figure 3c-d).

### Intermediate levels of disturbance displayed increased dissimilarity with time

**Fig. 3:**
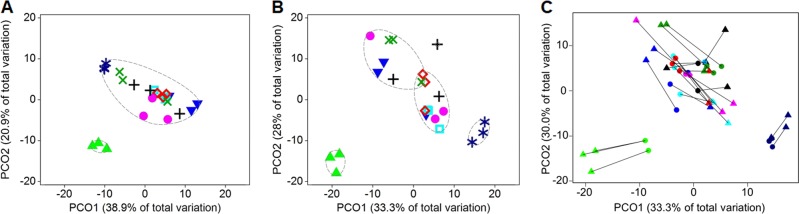
Community dissimilarity assessed by principal coordinates analysis (PCO) plots for all disturbance levels on T-RFLP datasets on days **a** 14 and **b** 35 of the study. Ovals with dashed lines represent 80% similarity calculated by group average clustering. Disturbance levels: L0 [light-green triangles], L1 [blue upside-down triangles], L2 [light-blue open squares], L3 [open red rhombuses], L4 [purple circles], L5 [black crosses], L6 [green x-symbols], and L7 [blue stars]. **c** Procrustes analysis on PCO at day 35 comparing metagenomics (circles) and T-RFLP (triangles) datasets. Lines unite data points from the same reactor (*n* = 24). Same colour palette as for disturbance levels. Tests comparing both methods were statistically significant (Supplementary Table 2). Intermediate treatments’ (L1–6) within-treatment dissimilarity increased with time. L0 and L7 clusters consistently displayed higher similarity after 14 days

To distinguish the effect of disturbance from temporal community dynamics (Fig. 1), community dissimilarity was assessed on the T-RFLP dataset at each time point by ordination analysis using principal coordinates analysis (PCO) (Fig. 3a, b), non-metric multidimensional scaling (NMDS), and canonical analysis of principal coordinates (CAP) with cluster similarity analysis (Supplementary Figure 4). The combination of constrained and unconstrained ordination methods allowed differentiating location from dispersion effects in community structure.^47^ L0 was consistently different in all ordination plots and L7 differed after 21 days, both with 0% misclassification error at all time points for CAP plots. Dispersion effects within intermediate levels were evident in the unconstrained ordination plots with higher differentiation of biological replicates after 35 days (Fig. 3b), coinciding with the production of nitrite and low levels of nitrate (Fig. 2d). Community differentiation was statistically significant from day 21 onwards as supported by PERMANOVA and PERMDISP (Supplementary Table 1).

### Metagenomics community analysis validates observations from fingerprint dataset

β-Diversity patterns observed from 16S rRNA gene amplicon T-RFLP data on day 35 were significantly similar to those from shotgun metagenomics data. A Mantel test on Bray–Curtis distance matrixes for both datasets (*n* = 24) yielded significant similarity (*r* = 0.73, *P* = 0.002). Procrustes tests of comparisons within ordination methods of PCO (Fig. 3c) and NMDS also yielded significant similarities for both datasets (*P* = 0.002, Supplementary Table 2). Multivariate PERMANOVA tests on the metagenomics dataset produced statistically significant results, but with significant heteroscedasticity as shown by PERMDISP (Supplementary Table 1). We resolved these mean–variance relationship concerns by running a general linear multivariate models (GLMMs) test to fit the data to a negative binomial distribution. Both residuals vs fitted and mean–variance plots supported the choice of a negative binomial distribution for the regression model (Supplementary Figure 5). The analysis of deviance of the regression rejected the null hypothesis of no difference between communities at different disturbance levels, independent of heteroscedasticity (*P* = 0.0149).

### Higher α-diversity for intermediately disturbed treatments and diversity-function correlations

**Fig. 4:**
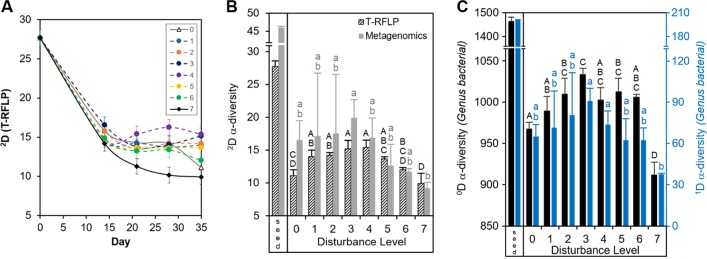
α-Diversity patterns. **a** Temporal dynamics of Hill number ^2^D for abundant OTUs, calculated from T-RFLP data across disturbance levels. **b** Hill number ^2^D calculated from T-RFLP (black dashed bars) and metagenomics (grey solid bars) data at days 0 (seed) and 35 (disturbance levels L0–L7). **c** Hill numbers ^0^D (black solid bars) and ^1^D (blue solid bars) from metagenomics data on days 0 (seed) and 35 (L0–L7). Values represent mean ± s.d. (*n* = 3). Characters above bars indicate Games–Howell post-hoc grouping

The observed patterns in α-diversity were time-dependent, as diversity decreased over time with respect to the initial sludge inoculum (Fig. 4a, T-RFLP dataset). Such a temporal decrease in diversity was higher at the extreme ends of the disturbance range, resulting in a parabolic pattern on day 35 (Fig. 4b, c). The final α-diversity pattern based on Hill number ^2^D was similar for both T-RFLP and metagenomics methods (Fig. 4b), although the latter showed higher variability. For the metagenomics dataset we also calculated the lower-order Hill numbers (^0^D, ^1^D) which give higher weight to less abundant operational taxonomic units (OTUs). They displayed the same parabolic pattern (Fig. 4c). Welch’s ANOVA tests were statistically significant for all Hill numbers (*P* < 0.01, *P* = 0.022 for ^2^D_metagenomics_). Additionally, there were strong significant correlations between α-diversity and ecosystem function (Supplementary Figure 6), focusing on the more robust estimators of microbial diversity ^1^D and ^2^D.^48^ Both ^1^D and ^2^D correlated positively with ammonia removal and nitrite generation (Supplementary Figure 6a-b), while ^2^D had a positive correlation with biomass (Supplementary Figure 6c) but a negative correlation with organic carbon removal (Supplementary Figure 6d).

### Null model analysis suggests different assembly mechanisms across disturbance frequencies

**Fig. 5:**
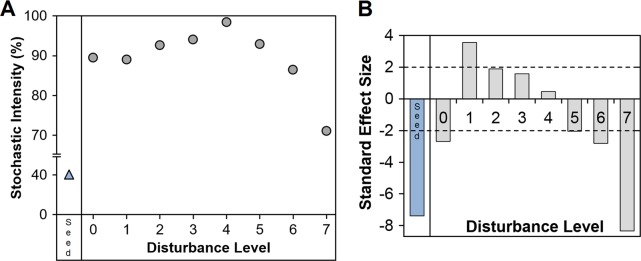
Influence of stochastic assembly mechanisms in bacterial communities as assessed by **a** stochastic intensity and **b** standard effect size (SES). Both metrics were calculated through null model analysis on the metagenomics genus-level dataset at days 0 (seed) and 35 (disturbance levels L0–L7). Each calculation involved all replicates of each treatment (*n*_seed_ = 2, *n*_L0–L7_ = 3) evaluated over 10,000 null model iterations. SES values closer to zero represent communities less deviant from the null expectation, implying stronger stochastic assembly processes. Overall, stochasticity was stronger for intermediate disturbance levels L2–L5 and also increased with respect to the sludge inoculum

To test if the observed changes in β-diversity (Figs 1a and 3, Supplementary Figure 4) were due to variations in the underlying stochastic and deterministic mechanisms or due to changes in α- and γ-diversity ratios (α:γ) alone,^49^ we employed a null model analysis from Kraft et al.^50^ on the bacterial genus-level metagenomics datasets on day 0 and day 35. The model estimated null β-diversity values after randomizing the location of each individual within the three independent reactors for each of the eight disturbance treatment levels, while keeping the total quantity of individuals per reactor, the relative abundance of each OTU, and the γ-diversity constant over 10,000 iterations. Under this model, stochastic assembly mechanisms were found to be higher for some intermediately disturbed levels (L2–L5) in terms of stochastic intensity (SI) and standard effect size (SES) values, which corresponded to communities less deviant from the null expectation (Fig. 5). SI was also higher at d35 with respect to the sludge inoculum (d0).

### Deterministic and stochastic patterns of assembly amongst different disturbance levels

Niche-structuring at both ends of the disturbance frequency range was suggested by community structure patterns and ecosystem function. The undisturbed (L0) and press-disturbed (L7) levels were distinct from each other as well as from the remaining intermediate levels, as supported by multivariate tests (both distance-based and GLMMs). The ordination plots and cluster analyses showed a clear separate clustering for the independent replicates of these two disturbance levels along the experiment, and particularly the constrained ordination plots displayed this with 0% misclassification error. Furthermore, the ecosystem function was clearly differentiated between L0 and L7, as well as being consistent across replicates at each level. We contend that the observed clustering is an indication that both the undisturbed and press-disturbed levels favoured deterministic assembly mechanisms, where the selective pressure due to unaltered succession (L0) or sustained toxic-stress (L7) promoted species sorting, resulting in similar community structuring among biological replicates over the course of the experiment.

Conversely, the communities from intermediately disturbed levels (L1–6) did not form distinct clusters for any particular level through the experiment. Within-treatment dissimilarity among replicates increased over time, with some replicates being more similar to those of other intermediate levels. Concurrently, ecosystem function parameters also displayed within-treatment variability for L1–6. For example, the conversion of ammonia to NO_X_ products, which was initially hampered when communities were still adapting to degrade 3-CA, was not the same across all equally handled independent replicates. The observed divergence across independent replicates is considered here as a strong indicator of stochasticity in community assembly. Additionally, the lower deviation for L2–L5 from expected β-diversity values estimated via null model analysis indicates a higher role of stochasticity at intermediate disturbance levels. Several processes might be promoting stochastic assembly, like strong feedback processes^51^ that are linked to density dependence and species interactions,^52^ priority effects,^53^ and ecological drift.^54^ Reactors within this study were designed as closed systems, hence stochastic dispersal processes^55^ could not affect community assembly.

We argue that there were different underlying stochastic–deterministic mechanisms operating in the resulting community assembly along the disturbance range of our study. Similarly, a study on groundwater microbial communities^18^ found through null model analysis that both deterministic and stochastic processes played important roles in controlling community assembly and succession, but their relative importance was time-dependent. The greater role of stochasticity we found on day 35 concurred with higher observed variability in the ecosystem function and structure among replicates for intermediately-disturbed levels. Likewise, previous work on freshwater ponds tracking changes in producers and animals^49^ found β-diversity (in terms of dissimilarity) increasing with stochastic processes. These observed patterns are also in accordance with ecological studies proposing deterministic and stochastic processes balancing each other to allow coexistence,^10^ with communities exhibiting variations in the strength of stabilization mechanisms and the degree of fitness equivalence among species.^9^ Thus, it is not sufficient to ask whether communities mirror either stochastic or deterministic processes,^8^ but also necessary to investigate the combination of such mechanisms that in turn explain the observed community structures along a continuum.^9^

### Diversity–disturbance patterns and trade-offs with function

We observed the highest α-diversity at intermediate levels as predicted by the IDH,^31^ both in terms of composition (^0^D) and abundances (^1^D, ^2^D). This finding is non-trivial in two aspects. First, Svensson et al.^32^ have shown that most studies find support for the IDH by using species richness (^0^D) rather than evenness or other abundance-related indices (like ^1^D and ^2^D). They suggested that low evenness at high disturbance levels could be caused by the dominance of a few disturbance specialists. Second, the use of richness for microbial communities is not reliable^48^ since it is heavily constrained by the method of measurement,^56^ which makes it hard to compare results from different studies using this metric. Additionally, for complex communities there is often a huge difference between the abundance of rare and abundant taxa. Hence, for microbial systems, it is reasonable to assess diversity in terms of more robust compound indices rather than richness, the reason why we focused on ^1^D and ^2^D for diversity-function analyses.

Importantly, the observed pattern in α-diversity was time-dependent and resulted in an IDH pattern after 35 days. Temporal dynamics were expected since the sludge community experienced an initial perturbation in all reactors after transfer from a wastewater treatment plant to our microcosm arrangement. For the sludge inoculum, this implied changes in reactor volume, frequency of feeding (continuous to batch), type of feeding (sewage to complex synthetic media), immigration rates (open to closed system), and mean cell residence time (low to high). This was a succession scenario in which communities had to adapt to such changes along with the designed disturbance array. For L0 and L7, ^2^D decreased over time in agreement with deterministically-dominated processes, probably because such levels represented the most predictable environments within our disturbance range. In contrast, intermediate levels either increased or maintained the same ^2^D over time (after an initial decrease within the first 2 weeks), seemingly a case where niche overlap promoted stochastic assembly.^8^ The emergence of an IDH pattern after time is coherent with findings in previous microcosm studies using synthetic communities of protists^57^ and freshwater enrichment microbial communities.^35^ Yet, none of these studies evaluated the relative importance of the underlying assembly mechanisms for the observed diversity dynamics.

Additionally, both ^1^D and ^2^D were positively correlated with nitrification and productivity, suggesting that higher community evenness favours functionality under selective pressure,^58^ but were negatively correlated with organic carbon removal. Thus, we cannot affirm that more diverse communities have better functionality without considering trade-offs. This supports the notion that higher α-diversity does not necessarily imply a “better” or “healthier” system.^56^ In addition to the observed changes in OTU diversity, there was an evident variation in ecosystem function along the disturbance range studied (Fig. 2c, d), a similar finding to that of previous studies with simpler planktonic communities.^59^

Functional trade-offs are expected under disturbance since organisms need to allocate resources normally used for other functions to recover after a disturbance.^60^ In our study, communities with higher biomass had lower organic carbon removal efficiencies, which together with the trade-offs described for nitrification, suggest the adoption of different community life-history strategies depending on the frequency of disturbance. The results presented here were all taxonomy-independent since our focus was on diversity, function, and mechanisms of community assembly (phylum-level community changes are provided as supplemental material Supplementary Figure 7). Taxonomy-independent approaches continue to be useful to describe diversity patterns and mechanisms of community assembly.^2,61^ However, it has been proposed that species’ traits can predict the effects of disturbance and productivity on diversity.^62^ Hence, further analysis of the different taxa and their genetic potential paired with the observed trade-offs in ecosystem function will aid in the understanding of potential life-history strategies^60^ and their relationship with community aggregated traits^63^ in the near future.

### Merging mechanisms of community assembly and alpha-diversity patterns: an intermediate stochasticity hypothesis

**Fig. 6:**
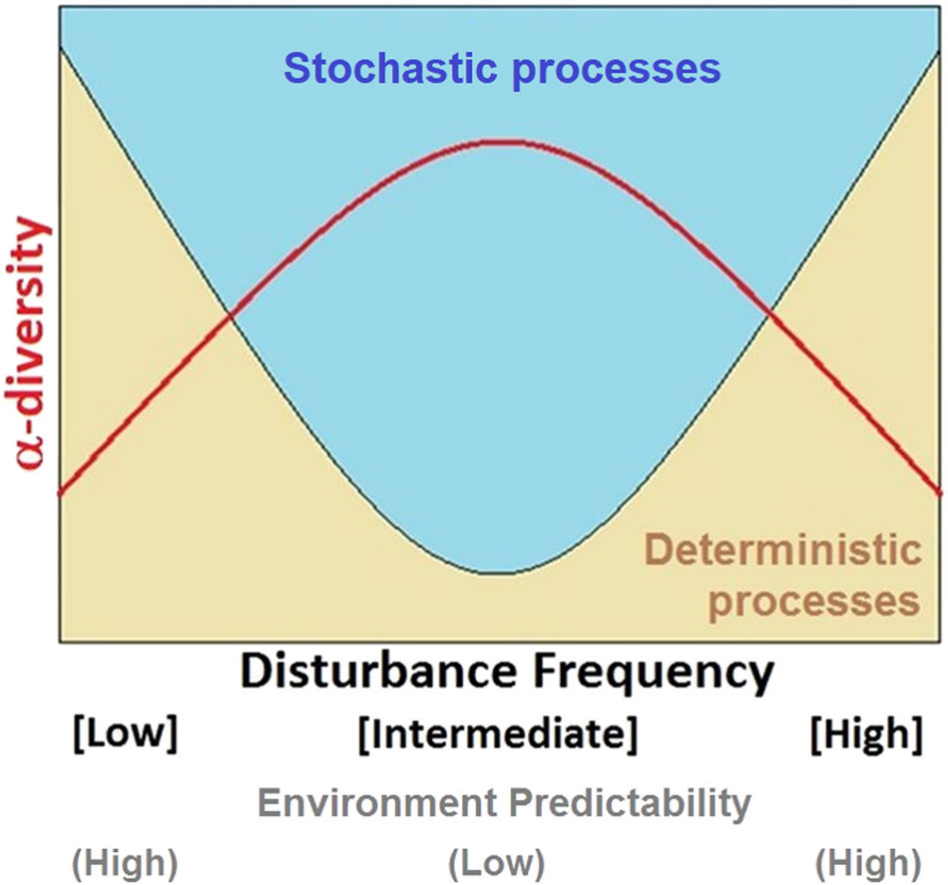
Intermediate stochasticity hypothesis (ISH) for community assembly under varying disturbances. Conceptual representation of the classic relationship between α-diversity and disturbance,^31^ including the effect of underlying stochastic and deterministic processes driving bacterial community assembly. When intermediate disturbance regimes result in less predictable environments, specialized traits would be less advantageous to taxa, and the stochastic equalization of competitive advantages would lead to higher α-diversity. On the contrary, extreme ends of the range where conditions are recurrent would select for adapted organisms whose dominance would result in a lower α-diversity

Knowing that the validity of the IDH is still under debate^37,38^ and that many different diversity–disturbance patterns have been reported,^28,30,33^ we asked whether there is a relationship between the peaked pattern in diversity observed and the underlying stochastic–deterministic processes of community assembly. Under purely stochastic processes, diversity should vary randomly as all species have equal fitness,^55^ unless some other mechanism acts to prevent this. It is recognized that, beyond empirical pattern description, an understanding of the underlying mechanisms is necessary to comprehend the outcomes of intermediate disturbance regimes.^30,40,64^ We hypothesize that higher α-diversity at intermediate disturbance frequencies is the result of weaker stabilizing mechanisms (niches), which are stronger at extreme ends of the disturbance range. Stochastic mechanisms will produce even assemblages (higher α-diversity) at intermediately disturbed levels, whilst infrequent or too-frequent disturbances will favour some species over others (lower α-diversity). We propose this idea as the intermediate stochasticity hypothesis (ISH, Fig. 6) and contend that it should hold particularly for compound α-diversity indices,^48^ since the underlying assembly mechanisms would affect taxa abundance distributions.

The ISH can be further portrayed by noting a key reasoning behind the IDH, namely, that a competition–colonization trade-off would lead to higher diversity at intermediate levels of disturbance.^31^ In the context of our study, which comprised a closed system, *colonization* would come from the low abundance taxa that have an opportunity to grow after different disturbance frequencies reduced the competitive ability of previously dominant taxa. Stochastic mechanisms of ecological drift could then play a critical role in shaping the emerging structure of microbial communities^3^ where random processes of birth, death, and reproduction can have an effect on which of these low abundance taxa will be more benefited as a result of intermediate disturbance frequencies. Drift could also lead to historical contingency and priority effects that are also stochastic,^53^ where taxa that occupy early the disturbance-opened niches could reduce the availability of resources to other taxa whose abundance will then be limited. Such reasoning could explain why, while higher α-diversity was found at intermediate levels of disturbance in our study, community structure and ecosystem function differed across identically treated replicates.

### Implications and concluding remarks

The implications of this study relate to both process engineering and environmental management. Sludge communities within wastewater treatment are not only model systems in microbial ecology,^65^ but also a key driver for water sanitation and the environmental impact of anthropogenic water discharges.^66^ Disturbances could promote stochastic assemblages of the sludge communities, which despite harbouring higher diversity could lead to variable overall ecosystem function. This could be the reason why after similar perturbations the process outcome differs, causing operational problems for water utilities.^67^ Furthermore, cases where disturbance temporally favours stochastic assembly could lead to a different final community after the perturbation,^27^ which could compromise the expected ecosystem function. More research is needed to identify such scenarios in practice.

We described how different frequencies of disturbance affected ecosystem function and bacterial community diversity and assembly in a closed microcosm bioreactors system. Communities were assessed through different molecular methods that nonetheless yielded very similar patterns. Furthermore, besides the wastewater treatment microbial community, other complex microbial systems (e.g., the gut microbiome) might display similar responses to disturbance. We argue that changes not only in diversity but also in the underlying deterministic–stochastic assembly mechanisms should be evaluated in studies of the effects of disturbance on such systems. For such an assessment, both replication and wide-enough disturbance ranges are key. Additionally, the ISH could be evaluated within open systems to include the effect of dispersal processes. This calls for more studies in microcosm^45,68^ and mesocosm settings, as well as meta-analysis from full-scale application studies.

## Methods

### Experimental design

We employed sequencing batch microcosm bioreactors (20-mL working volume) inoculated with activated sludge from a full-scale plant and operated for 35 days. The daily complex synthetic feed (adapted from Hesselmann et al.^69^) included toxic 3-CA at varying frequencies. Eight levels of disturbance were set in triplicate independent reactors (*n* = 24), which received 3-CA every day (press-disturbed), every 2, 3, 4, 5, 6, or 7 days (intermediately-disturbed), or never (undisturbed). Level numbers were assigned from 0 to 7 (0 for no disturbance, 1 to 7 for low to high disturbance frequency, Supplementary Figure 1). Ecosystem function, in the form of process performance parameters at the end of a cycle, was measured weekly in accordance with Standard Methods^70^ where appropriate, and targeted soluble chemical oxygen demand (COD), nitrogen species (ammonium, nitrite, and nitrate ions) and 3-CA, and volatile suspended solids (VSS). On the initial day and from the second week onwards, sludge samples (2 mL) were collected weekly for DNA extraction.

### 16S rRNA gene amplicon fingerprinting and processing

DNA extracted from all sludge samples (*n* = 99) was analysed by T-RFLP of the 16S rRNA gene using the 530F–1050R primer set targeting V4–V5 regions. The PCR program included initial denaturation at 95 °C for 10 min, followed by 30 cycles of denaturation (95 °C, 1 min), annealing (58 °C, 30 s) and extension (72 °C, 1 min), and final extension at 72 °C for 7 min. Purified DNA products were digested using the BsuRI (HaeIII) enzyme through incubating at 37 °C for 16 h. Enzyme inactivation was performed at 80 °C for 20 min. Digested DNA was subjected to T-RFLP on an ABI 3730XL DNA analyser. Sequence alignment files from T-RFLP runs were assessed for quality control and pre-processed using the software GeneMapper v.5 (Applied Biosystems).^71^ Peak areas were normalized to the total area per sample^72^ and de-noised using a conservative fluorescence threshold of 200 units.^73^

### Metagenomics sequencing and reads processing

Purified genomic DNA from sludge samples on d0 (inoculum) and d35 (*n* = 24) were subjected to metagenomics sequencing at the SCELSE sequencing facility (Singapore). Library preparation was performed according to Illumina’s TruSeq Nano DNA Sample Preparation protocol. Libraries were sequenced in one lane on an Illumina HiSeq 2500 sequencer in rapid mode at a final concentration of 11 pM and a read-length of 250 bp paired-end. Around 173 million paired-end reads were generated in total and 7.2 ± 0.7 million paired-end reads on an average per sample. Illumina adaptors, short reads, low quality reads or reads containing any ambiguous base were removed using *cutadapt* (–m 50 –q 20 - --max-n 0, v.1.11).^74^ Taxonomic assignment of metagenomics reads was done following the method described by Ilott et al.^75^ High quality reads (99.2 ± 0.09% of the raw reads) were randomly subsampled to an even depth of 12,395,400 for each sample prior to further analysis. They were aligned against the NCBI non-redundant (NR) protein database (March 2016) using DIAMOND (v.0.7.10.59) with default parameters.^76^ The lowest common ancestor approach implemented in MEGAN Community Edition v.6.5.5^77^ was used to assign taxonomy to the NCBI-NR aligned reads with the following parameters: maxMatches = 25, minScore = 50, min Support = 20, paired = true. On average, 48.2% of the high-quality reads were assigned to cellular organisms, from which in turn 98% were assigned to the bacterial domain. Adequacy of sequencing depth was corroborated with rarefaction curves at the genus taxonomy level (Supplementary Figure 8) using the *rarefy* function of the *vegan* R-package (v.2.5-2). We did not include genotypic information as it was outside the scope of this study, but will do so in future investigations arising from this work.

### Microbial community analysis and statistical tests

All reported *P*-values for statistical tests in this study were corrected for multiple comparisons using a false discovery rate (FDR) of 10%.^78^ Community structure was assessed by a combination of ordination methods (PCO, NMDS, CAP) and multivariate tests (PERMANOVA, PERMDISP)^79^ on Bray–Curtis dissimilarity matrixes constructed from square-root transformed normalized abundance data using PRIMER (v.7). Additionally, GLMMs, which deal with mean–variance relationships,^80^ were employed using the *mvabund* R-package^81^ fitting the metagenomics dataset to a negative binomial distribution, to ensure that the observed differences among groups were due to disturbance levels and not heteroscedasticity. The 500 most abundant genera (97% of total assigned reads abundances) were employed to ensure random distribution of residuals fitted in the model. Significance was tested using the *anova* function in R with PIT-trap bootstrap resampling (*n* = 999).^82^ Hill diversity indices^83^ were employed to measure α-diversity as described elsewhere,^48,84^ and calculated for normalized non-transformed relative abundance data.

### Comparison between metagenomics and T-RFLP community datasets

Mantel and Procrustes tests^85^ were applied to compare metagenomics and T-RFLP datasets from all bioreactors on day 35 (*n* = 24, subsample of the full T-RFLP dataset). Such an approach is valid for the questions asked in this study, since comparisons between NGS and fingerprinting techniques support the use of T-RFLP to detect meaningful community assembly patterns and correlations with environmental variables,^61^ and such patterns can be validated by NGS on a subset of the fingerprinting dataset.^2^

Bray–Curtis dissimilarity matrixes were computed using square root transformed T-RFLP data and bacterial genus-level taxa tables generated using a metagenomics approach. Mantel tests were then used to determine the strength and significance of the Pearson product–moment correlation between complete dissimilarity matrices. Procrustes tests (PROTEST) were also employed as an alternative approach to Mantel tests in order to compare and visualize both matrices on PCO and NMDS ordinations. The resultant m2-value is a statistic that describes the degree of concordance between the two matrices evaluated.^86^ All these statistical tests were performed using the *vegan* R-package (functions: *procuste*, *mantel*, *metaMDS*, *vegdist*).

### Null model analysis on diversity

To disentangle the roles of stochastic and deterministic processes as drivers of change in β-diversity it is necessary to incorporate a statistical null model in the analysis,^87^ which assumes that species interactions are not important for community assembly.^88^ We employed a null model approach originally applied to woody plants^50^ and more recently to microbial communities.^18^ The model defines β-diversity as the β-partition $(\beta=1-\overline{\alpha}/\gamma)$^89^ and takes into account both composition and relative abundances. To adapt it to handle microbial community data, we considered species as OTUs (genus taxonomic level) and each individual count as one read within the metagenomics dataset. The model randomizes the location of each individual within the three independent reactors for each of the eight disturbance treatment levels, while maintaining the total quantity of individuals per reactor, the relative abundance of each OTU, and the γ-diversity. We applied it to the metagenomics datasets from d0 and d35.

Each step of the null model calculates expected mean α-diversities for each disturbance level and then estimates an expected β-partition. After 10,000 repetitions, the mean and standard deviation of the distribution of random β-partitions for each disturbance level are calculated. The output of this model is a β-deviation or SES, which is the observed β-diversity (*β*_obs_) minus the mean of the null distribution of β-diversity values $(\overline{\beta_{\mathrm {exp}}})$, divided by the standard deviation of this distribution (*σ*_exp_), SES = $(\beta_{\mathrm {obs}}-\overline{\beta_{\mathrm {exp}}})/\sigma_{\mathrm {exp}}$. We further calculated the SI as the difference between the observed and mean expected β-diversities divided by the observed β-diversity, SI = $(\beta_{\mathrm {obs}}-\overline{\beta_{\mathrm {exp}}})/\beta_{\mathrm {obs}}$.

### Reporting summary

Further information on experimental design is available in the Nature Research Reporting Summary linked to this article.

## Supplementary information

Supplementary information

Reporting Summary

## Data Availability

DNA sequencing data are available at NCBI BioProjects with accession number: 389377. See Supplementary Methods for details on sludge inoculum collection, complex synthetic wastewater preparation, scheme for ecosystem function measurement and sludge collection, chemical analysis, DNA extractions, 16S rRNA gene community fingerprinting, metagenomics library preparation and sequencing, multivariate analyses, alpha-diversity indices, univariate analysis of variance and correlation tests, and null model analysis. Ecosystem function data, R-script for the null model analyses, T-RFLP raw data, and all other relevant data can be publicly accessed on FigShare (10.6084/m9.figshare.7369964).

## **Supplementary information**

**Supplementary information** accompanies the paper on the *npj Biofilms and Microbiomes* website (10.1038/s41522-019-0079-4).
