# Supplementary material for: Frequency of disturbance alters diversity, function, and underlying assembly mechanisms of complex bacterial communities
Source: NPJ Biofilms Microbiomes. 2019 Feb 11;5:8. doi: 10.1038/s41522-019-0079-4 (PMC6370796; doi:10.1038/s41522-019-0079-4)
Supplement: Supplementary file 1 — Supplementary information [file 41522_2019_79_MOESM1_ESM.pdf]

Supplementary Information

Frequency of disturbance alters diversity, function, and underlying assembly mechanisms of complex bacterial communities

Authors: Ezequiel Santillan[1,2], Hari Seshan[1,2†], Florentin Constancias[1], Daniela I. Drautz-Moses[1], and Stefan Wuertz[1,2,3*]

Affiliations:

[1]Singapore Centre for Environmental Life Sciences Engineering, Nanyang Technological University, 637551, Singapore.

[2]Department of Civil and Environmental Engineering, University of California, Davis, CA 95616, U.S.A.

[3]School of Civil and Environmental Engineering, Nanyang Technological University, 639798, Singapore.

*Correspondence to: Stefan Wuertz, swuertz@ntu.edu.sg.

†Present address: Brown and Caldwell, 9665 Chesapeake Drive, Suite 201, San Diego CA 92123, U.S.A.

Supplementary Methods

Sludge inoculum collection

Sludge inoculum was collected from the activated sludge tanks of a full-scale water reclamation plant (WRP) in Singapore. Flow rate Q = 200,000 m^3 d^{-1}, T = 29-31°C, hydraulic retention time (HRT) = 8 h, solids retention time (SRT) = 6 d. The WRP receives a mix of residential, commercial and industrial wastewater as its influent. Activated sludge was collected in 20-L containers and immediately transported to the lab. The suspension was manually mixed by shaking the closed container thoroughly before transferring half of it to a 10-L vessel that was stirred using a magnetic stir plate to ensure homogeneity. Samples of 20 mL were transferred to 50-mL tubes (n =24) (Eppendorf), which served as bioreactors in a microcosm setup. About 30 min of settling time was allowed and 10 mL of supernatant was removed and replaced with 10 mL of synthetic wastewater with or without 3-CA as described below. On the first day a mix of synthetic wastewater (adapted from Hesselmann *et al.*[1]) with 3-CA was added to reactors for levels 1 to 7, while level 0 reactors received synthetic wastewater without 3-CA. Additionally, eight reactors served as abiotic controls with only synthetic wastewater and 3-CA (no sludge), and were maintained to evaluate 3-CA loss due to any abiotic processes like wall adsorption.

Complex synthetic wastewater preparation

The complex synthetic wastewater contained the following (nominal concentration in mg L^{-1} mixed liquor after feeding): yeast extract (67.5), soy peptone (60), meat peptone (60), casein peptone (90), sodium acetate (112.5), dextrose (45), urea (15), ammonium bicarbonate (90), ammonium chloride (169), disodium hydrogen phosphate (720), potassium dihydrogen phosphate (130), calcium chloride dihydrate (10.5) and magnesium sulfate heptahydrate (112.5). The medium also contained 2 mL L^{-1} of the unaltered trace element stock[1]. The first six components contributed to the chemical oxygen demand (COD), amounting to about 590

(±15.4) mg L^{-1}in mixed liquor, the next three contributed to about 92 (±2.5) mg-N L^{-1} (in the form of ammonium compounds), and the phosphates were used to buffer the medium and maintain a pH of around 7.5 to facilitate the nitrification process. The medium for disturbed levels also included 140 mg L^{-1} of 3-CA that resulted in 70 mg L^{-1} in the mixed liquor (red squares in Fig. S1), a level proven to have significant effects on sludge bioreactors in previous experiments[2]. Whenever 3-CA was added the remaining organic carbon compounds were reduced proportionally to maintain a constant total COD. The medium was autoclaved after preparation to avoid contamination, and 3-CA was added under sterile conditions after autoclaved medium had cooled down. All reactors were capped and incubated until the following day in a shaking incubator at 30°C, the prevailing water temperature for wastewater treatment plants in Singapore.

Scheme for ecosystem function measurement and sludge collection

After each cycle (24 h) all the tubes were removed from the incubator and allowed to settle for 30 min, after which 10 mL of "effluent" supernatant liquid was removed and replaced aseptically with 10 mL of fresh synthetic medium, resulting in a 48-h HRT. Every seven days process performance data (COD, [NH_4^+], [NO_2^-], [NO_3^-], [3-CA]) were generated from effluent samples, and from the second week, sludge samples (2 mL) were collected for DNA extraction (Fig. S1). Effluent samples were filtered through a 0.2-μm pore size filter and the filtrate was stored at 4°C for less than one week prior to chemical analyses. Aliquots of sludge samples were flash frozen in liquid nitrogen immediately after collection and stored at -80°C for a maximum of four weeks before molecular analysis. On the final day of the experiment, all the remaining sludge was employed for gravimetric TSS and VSS measurements in accordance with Standard Methods[3]. The sludge collection scheme resulted in an SRT of 87.5 days. We purposely aimed for a long SRT to avoid any wash out and keep our microbial seed-bank to allow rare taxa to occupy niches potentially generated by disturbance events. The duration of

the experiment was set to 35 days due to clear signs of changes in ecosystem function across disturbance levels.

Chemical analysis

Water quality parameters were measured in accordance with Standard Methods[3] and targeted soluble COD (Standard Methods 5220 D) and nitrogen species (ammonium, nitrite, and nitrate ions) using spectrophotometric tests (Hach) and Ion Chromatography (Standard Methods 4500-NH3 for ammonium; 4110 B for nitrate and nitrite). 3-CA was measured on a Shimadzu Prominence High Pressure Liquid Chromatography (HPLC) system (Shimadzu) equipped with a UV-VIS PDA detector using an Ascentis C18 5-μm column (Sigma-Aldrich). An isocratic 50:50 Water:Methanol solvent was used at a flow rate of 0.3 mL min^{-1}, and 3-CA peaks were identified and measured at 199, 237 and 286 nm.

DNA extractions

Genomic DNA was extracted from about 500 μL of sludge using the FastDNA Spin Kit for Soil and the FastPrep Instrument (MP Biomedicals) with modifications to the manufacturer's protocol to increase DNA yield. The first modification involved performing four lysis cycles in the FastPrep instrument instead of one, with two minutes of rest in between each cycle, during which the samples were placed on ice[4]. The second modification involved eluting DNA from the spin column using nuclease-free water (Qiagen) that had been pre-heated to 55°C, followed by incubation of the columns in elution water at 55°C for five minutes before the final centrifugation. Extracted DNA was quantified using both NanoDrop 2000c and Qubit 3.0 fluorometer (both ThermoFisher Scientific).

16S rRNA gene community fingerprinting

DNA extracted from sludge samples was analysed by Terminal Restriction Fragment Length Polymorphism (T-RFLP) of the 16S rRNA gene using the restriction enzyme BsuRI

(HaeIII) (Fermentas, ThermoFisher Scientific), which was selected on the basis of good reproducibility and ability to produce a high number of defined peaks from samples on previous experiments[5,6]. T-RFLP and other fingerprinting techniques (e.g., DGGE, ARISA) are used by molecular ecologists to characterize and compare the composition and diversity of microbial communities[7-17]. Although T-RFLP has a limited resolution compared to next generation sequencing (NGS)[18,19], comparisons between NGS and fingerprinting techniques support the use of T-RFLP to detect meaningful community assembly patterns and correlations with environmental variables[17,20].

The bacterial 16S rRNA gene was first amplified from the extracted DNA using a primer set that included forward primer 530F (S-D-Bact-0515-a-S-16, 5'-GTGCCAGCMGCNGCGG-3')[21] labelled with fluorophore 6FAM at the 5' end, and reverse primer 1050R (S-D-Bact-1050-a-A-16, 5'-ACGACAGCCATGCANC-3')[22] labelled with fluorophore ROX at the 5' end. This primer set targeted the V4-V5 regions of the gene, generating a 550 bp amplicon (including primers), and was used to amplify each DNA extract in triplicate 50-μL PCR reactions. Each 50-μL PCR reaction contained 25 μL of ImmoMix reagent (Meridian Bioscience), 1 μL of each primer (1/10 diluted from manufacturer stock with nuclease-free water), about 300 ng of DNA extract, and nuclease-free water. The PCR program included an initial denaturation step at 95°C for 10 min, followed by 30 cycles of denaturation (95°C, 1 min), annealing (58°C, 30 s) and extension (72°C, 1 min). A final extension was carried out at 72°C for 7 min. The triplicate PCR amplicons from each sample were then pooled and purified using the QIAquick PCR purification kit (Qiagen, Venlo), with a slightly altered protocol in which the final elution was performed on nuclease-free water pre-heated to 55°C and incubated for five min at 55°C before the final centrifugation. This was found to increase the final DNA yield. Amplified DNA was quantified using Qubit, whereas gel electrophoresis was used to assess the quality of DNA bands after purification and to check that there was no

amplification among negative controls. The purified amplicons were then separately subjected to enzyme digestion. The digestion reaction mixture was prepared in 32-μL reactions according to the enzyme manufacturer's recommended protocol using about 10 μL of purified PCR product. The enzyme digestion was carried out by incubating at 37°C for 16 h. Enzyme inactivation was performed at 80°C for 20 minutes. This digested DNA was subjected to T-RFLP on an ABI 3730XL DNA analyser (Applied Biosystems). All samples were processed in the same run to avoid batch effects. Each T-RFLP reaction was carried out using 1 μL of digested DNA from the previous step along with 20 μL of Hi-Di Formamide (Applied Biosystems) and 1 μL of internal size standard GeneScan-600LIZ (Applied Biosystems). While each sample-primer set combination was subjected to enzyme digestion in single reactions, T-RFLP was performed in triplicate from each of these single enzyme digestion products (technical replicates). Sequence alignment files from T-RFLP runs generated in ".fsa" format by the ABI 3730XL analyser were assessed for quality control and pre-processed using the software GeneMapper v.5 (Applied Biosystems)[23]. Peak sizes were inspected manually and re-binned if necessary, and peak areas were normalized to the total area per sample[24]. De-noising of peak areas was carried out as described in Abdo *et al.*[25] using a conservative fluorescence threshold of 200 fluorescence units. Separate profiles from both dyes (6-FAM and ROX) were then combined into a single profile per sample replicate. The peak areas from triplicate T-RFLP runs (three technical replicates per independent sample) were inspected by NMDS ordination to detect and remove outliers among technical replicates and then averaged to produce one final profile per sample[25]. Only two outliers were removed after NMDS inspection from a total of 297 T-RFLP technical replicates corresponding to the 99 samples analysed.

Metagenomics library preparation and sequencing

Genomic DNA from sludge samples was cleaned using the Genomic DNA Clean & Concentrator kit (Zymo Research Corp) following the protocol from the manufacturer. Library

preparation and sequencing was carried at the SCELSE sequencing facility (Singapore). Prior to library preparation, the quality of the DNA samples was assessed on a Bioanalyzer 2100, using a DNA 12000 Chip (Agilent). Sample quantitation was carried out using Invitrogen's Picogreen assay. Library preparation was performed according to Illumina's TruSeq Nano DNA Sample Preparation protocol using a LT Sample Preparation Kit. DNA samples were sheared on a Covaris E220 to ~450bp, following the manufacturer's recommendation, and uniquely tagged with one of Illumina's barcodes (provided in the library preparation kit) to allow pooling of libraries for sequencing. The finished libraries were quantitated using Invitrogen's Picogreen assay and the average library size was determined on a Bioanalyzer 2100, using a DNA 7500 chip (Agilent). Library concentrations were then normalized to 4nM and validated by qPCR on a ViiA-7 real-time thermocycler (Applied Biosystems), using the KAPA Illumina Library Quantification Kit (Kapa Biosystems, Roche). The libraries were then pooled at equimolar concentrations and sequenced in one lane on an Illumina HiSeq2500 sequencer in rapid mode at a final concentration of 11pM and a read-length of 250bp paired-end.

Multivariate analyses

The experimental design was purposely balanced to allow for accurate hypothesis testing during statistical analysis, because we expected that stochastic assemblages might lead to heteroscedasticity among replicates[26]. For assessing similarity between microbial communities, ordination methods and clustering analysis were employed using normalized abundances data, which had been square root transformed to reduce the weight of the most abundant OTUs[13,27]. The analysis was made using PRIMER (v.7) with PERMANOVA+ (primer-e Ltd, Ivybridge, UK).

Bray-Curtis similarity matrices were used to make unconstrained ordination plots of Non-metric Multidimensional Scaling (NMDS)[28] and Principal Coordinates analysis (PCO)[20],

constrained ordination plots of Canonical Analysis of Principal coordinates (CAP)[29], and CLUSTER analysis[30]. Each of these multivariate techniques can employ Bray-Curtis (dis)similarities as input measurements[29] and provide a different perspective of community assembly, thus a combination of them can help visualize patterns that otherwise would be masked if using only one[29,31]. NMDS was performed with up to 50 iterations producing convergent solutions in two dimensions, where the goodness of each fit was assessed using Shepard plots and the stress value between the two-dimensional and high-dimensional dissimilarities. Values of stress <0.2 are normally a good depiction of similarity rankings[28]. PCO plots included percentage of variability explained by each axis. Accuracy of CAP plots was assessed by analysing misclassification errors by the leave-one-out method[29]. CLUSTER analysis used the Group Average method and included SIMPROF to test for significant differences between branches[30].

Permutational multivariate analysis of variance (PERMANOVA) was employed to test the null hypothesis that there were no statistically significant differences in community assembly among disturbance levels and temporally throughout the study[26]. Factors were considered fixed. Homogeneity of multivariate dispersions among groups was tested by PERMDISP[32]. P-values were calculated using 9,999 permutations.

Alpha diversity indices

Hill diversity indices[33] were employed to measure α-diversity as described elsewhere[34,35], and calculated for normalized, non-transformed relative abundance data. Hill numbers can provide useful information on the degree of change among communities[19,34,35], but underestimate ^{0}D and ^{1}D if calculated from fingerprinting data[18,19]. It was previously suggested that community composition rather than richness (^{0}D) is relevant for evaluating specific microbial processes, and ^{1}D and ^{2}D are preferred to quantify and compare microbial diversity, where ^{2}D can be estimated more robustly[35,36]. Thus, we focused on ^{2}D which is sensitive to

changes in abundances of common OTUs[19,33], calculated from both metagenomics and T-RFLP datasets. Additionally, ^{0}D and ^{1}D were obtained from the metagenomics dataset only, with the ^{1}D index giving a higher weight to less abundant (or rare) species compared to ^{2}D. These Hill numbers are calculated as shown below in equations [1] and [2], which also show their relationship with the often employed Shannon (H') and Simpson (λ) entropies, where p_i is the abundance proportion of an ith OTU (i.e. genus for metagenomics and TRFs for T-RFLP datasets) and R is the total number of OTUs (or richness, R = ^{0}D).

$$^{1}D = exp(-\sum_{i=1}^{R} p_i \cdot ln(p_i)) = e^{H'} \qquad [1]$$

$$^{2}D = \frac{1}{\sum_{i=1}^{R} p_i^2} = \frac{1}{\lambda} \qquad [2]$$

Univariate analysis of variance and correlation tests

Statistical univariate tests of Welch's ANOVA and Games-Howell post hoc analyses, both robust against non-normality and heteroscedasticity, were carried out on Hill α-diversity indices using SPSS v.25 (IBM Corp.). Monotonic correlations between process variables and diversity indices were assessed with SPSS using Spearman's rho (ρ). Reported correlations are only those verified to be significant after adjustment to a False Discovery Rate (FDR) of 10%[37].

Null model analysis

Metagenomics sample data were normalized to 100,000 reads (equivalent to 100,000 model individuals) for the null model analysis. In this manner the number of total individuals to be shuffled on each model iteration was reduced by one order of magnitude, while only reducing the γ-diversity of the overall dataset by three OTUs (1,504 genus-level assigned OTUs after normalization from an initial total of 1,507). This allowed us to increase the iterations of the model to 10,000 to enhance the calculation of the mean ($\overline{\beta_{exp}}$) and standard deviation (σ_{exp}) of the null distribution of β-diversity values.

The null model analysis from Kraft *et al.*[38] was previously employed for studies in groundwater microbial communities by Zhou *et al.*[39]. Our application of such model differed in two ways. First, we did not calculate pair-wise β-deviations and rather used all replicates available per treatment, since pair-wise comparisons introduce autocorrelation and bias to the output of the original method[38]. Second, we did not use Jaccard dissimilarities which are presence-absence based (and thus not reliable for microbial community analysis), but assessed the differences in relative abundances instead, as originally proposed by Kraft *et al.*[38].

Supplementary Figures

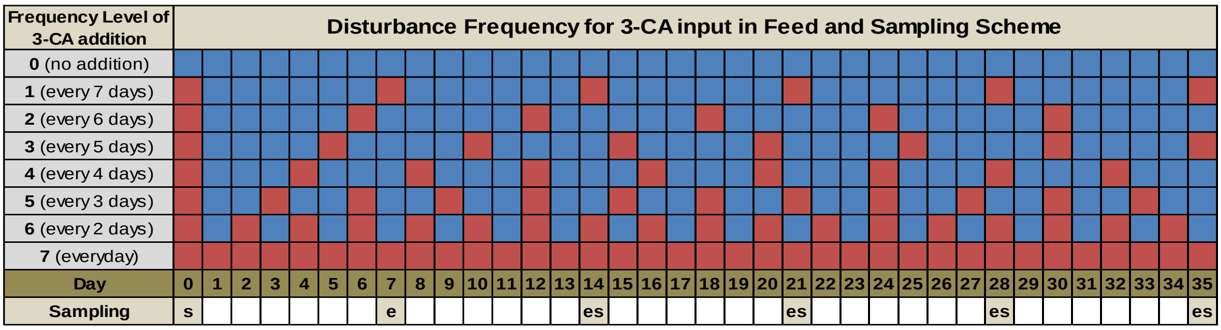

Supplementary Figure 1 Schematic representation of the experimental design and timeline. Blue squares represent no 3-chloroaniline (3-CA) in the feed and red squares indicate 3-CA addition for that particular day. Disturbance levels were numbered from 0 (no disturbance) to 7 (maximum disturbance frequency, press-disturbance). Effluent sampling for measurement of performance indicators is denoted by “e”, sludge sampling by “s”, and “es” indicates both effluent and sludge sampling.

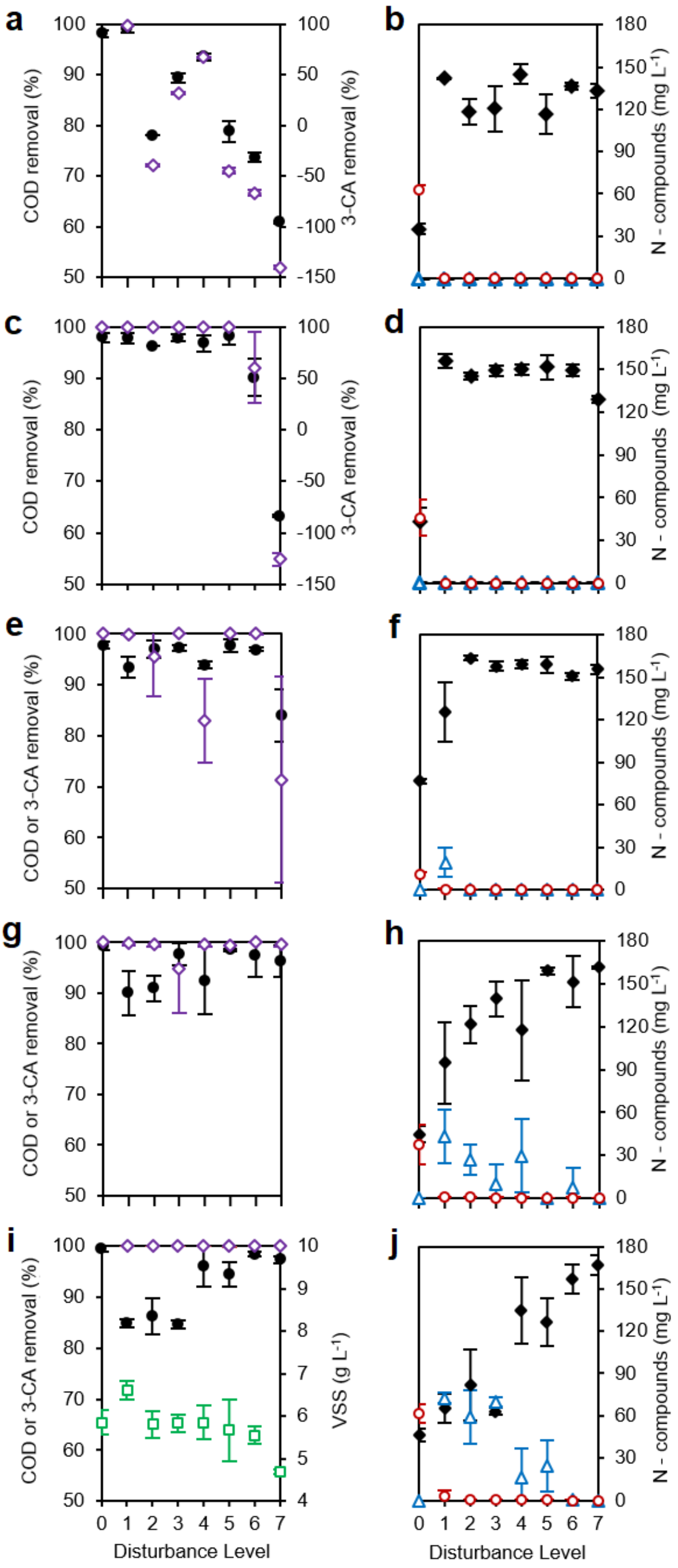

a
b
c
d
e
f
g
h
i
j
COD removal (%)
3-CA removal (%)
COD or 3-CA removal (%)
N - compounds (mg L-1)
VSS (g L-1)
Disturbance Level

Supplementary Figure 2 Process performance across disturbance levels and time. (**a**, **c**, **e**, **g**, **i**) Percentage of organic carbon as chemical oxygen demand (COD, ●) and 3-CA (◇) removal for all levels as measured weekly (negative values represent accumulation). (**i**) Biomass as volatile suspended solids as measured on day 35. (VSS, □). (**b**, **d**, **f**, **h**, **j**) Concentration of ammonium (◆), nitrite (△), and nitrate (○) as nitrogen for all levels as measured weekly. Data from days 7 (**a-b**), 14 (**c-d**), 21 (**e-f**), 28 (**g-h**) and 35 (**i-j**) of the study. Mean ± s.d. (n = 3) are shown. Panels from Fig. 2 (main text) are also included here to facilitate the interpretation on function temporal dynamics. Overall, process performance parameters indicate temporal changes and trade-offs in ecosystem function, increase in functional variability for intermediately disturbed levels (L1-6), and function differentiation for L0 and L7.

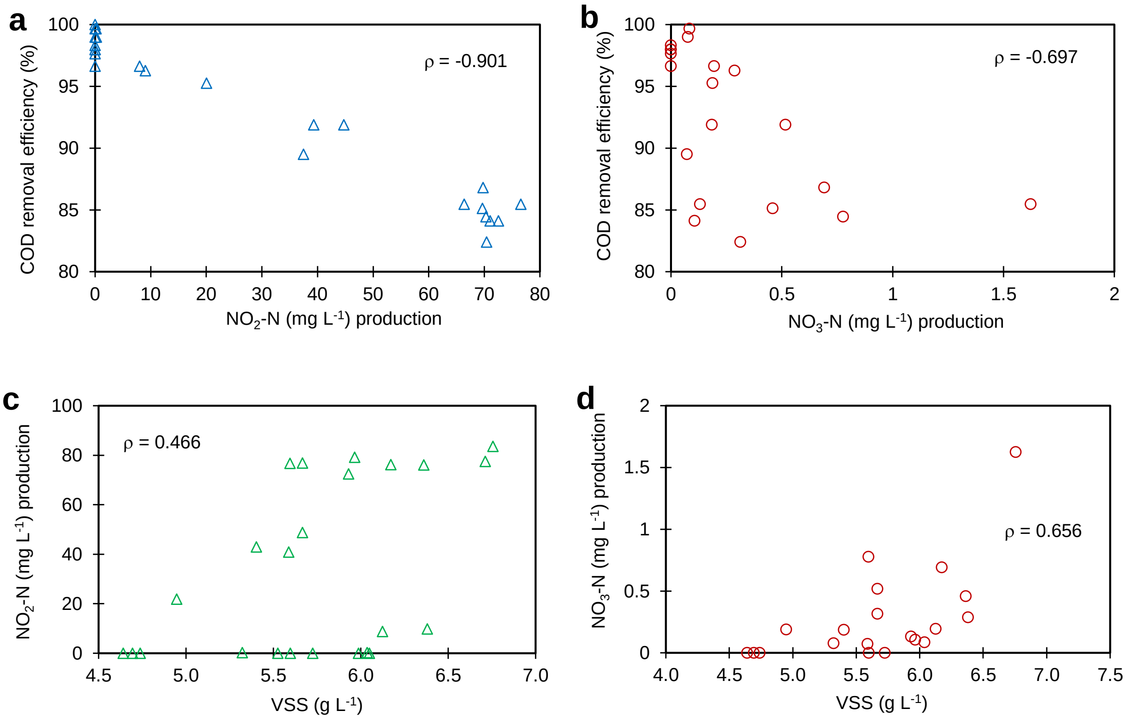

Supplementary Figure 3 Spearman's correlations of process performance indicators. Variation of organic carbon (COD, chemical oxygen demand) removal with (**a**) NO_2–N and (**b**) NO_3–N production. Variation of (**c**) NO_2–N and (**d**) NO_3–N production, as a function of biomass (VSS, volatile suspended solids). Data for all treatments on day 35. Only significant correlations after 10% FDR correction are reported. Correlation coefficients (ρ) are included. Plot (**b**) does not include data from Level 0, which consistently degraded COD and completely nitrified ammonia, so as to evaluate the correlation between COD removal and NO_3-N production within disturbed levels only. Plot (**d**) does not include data from Level 0, so as to evaluate the correlation between NO_3-N production and VSS within disturbed levels only. Overall, these correlations highlight trade-offs in ecosystem function.

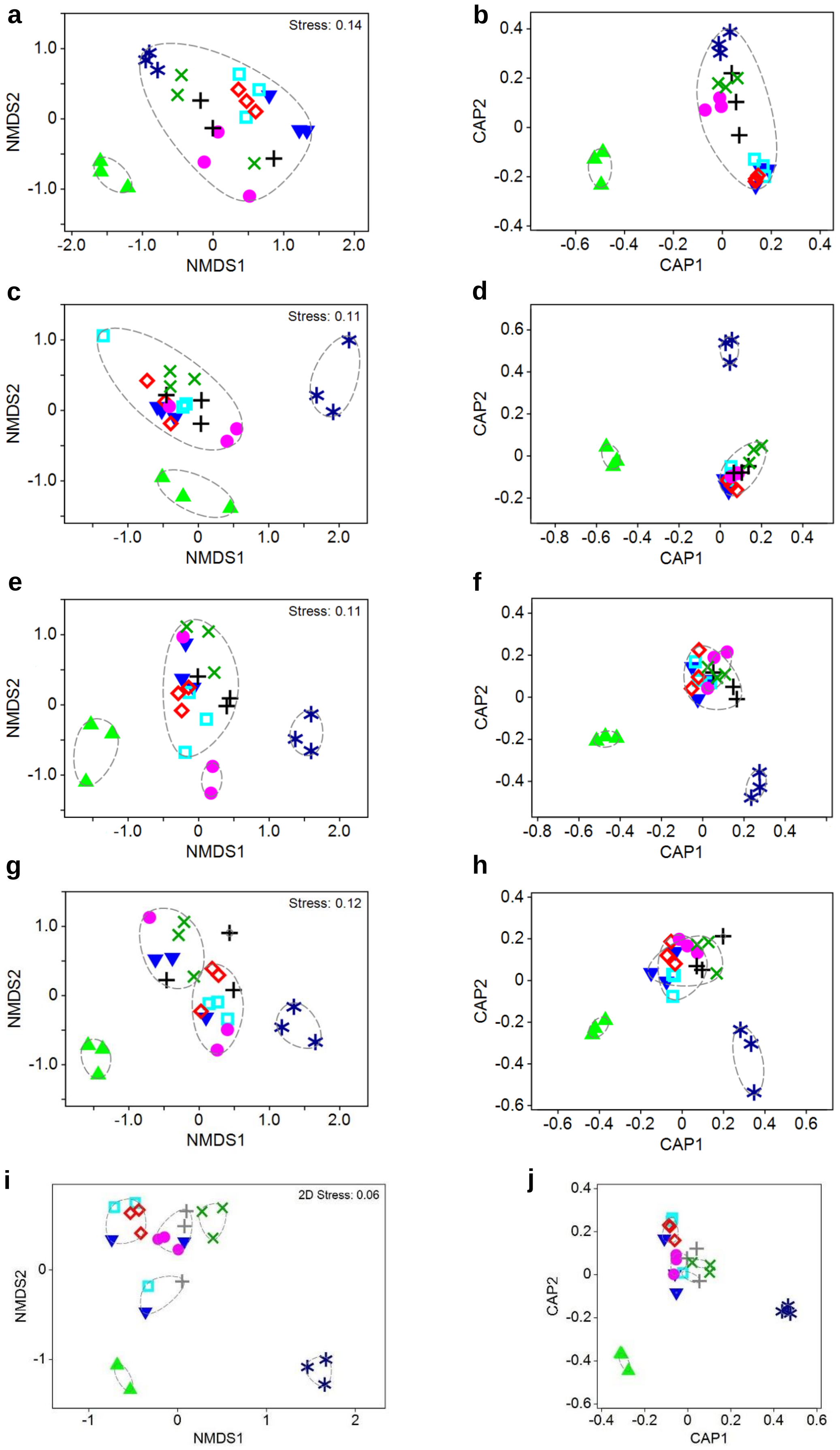
a
Stress: 0.14
NMDS2
NMDS1
b
CAP2
CAP1
c
Stress: 0.11
d
e
Stress: 0.11
f
g
Stress: 0.12
h
i
2D Stress: 0.06
j

Supplementary Figure 4 Unconstrained and constrained ordination plots with overlaid cluster analysis for different time points. Community assemblage as assessed by the unconstrained ordination method of Non-Metric Multidimensional Scaling (NMDS) for all disturbance levels on days (**a**) 14, (**c**) 21, (**e**) 28, and (**g**, **i**) 35 of the study; and community assemblage assessed by the constrained ordination method of Canonical Analysis of Principal coordinates (CAP) plots on days (**b**) 14, (**d**) 21, (**f**) 28, and (**h**, **j**) 35. Stress value is indicated for each NMDS plot. Disturbance levels: L0[▲], L1[▼], L2[◻], L3[◇], L4[●], L5[+], L6[×], and L7[✱]. Plots based on Bray-Curtis dissimilarity matrixes from T-RFLP (**a-h**) and metagenomics genus-level (**i-j**) data. Ovals with dashed lines represent 80% (**a-h**) and 94% (**i-j**) similarity calculated by group average clustering. Altogether, these plots illustrate increasing dispersion effect over time for intermediately disturbed treatments (L1-6), and differentiation of extreme treatments (L0 and L7).

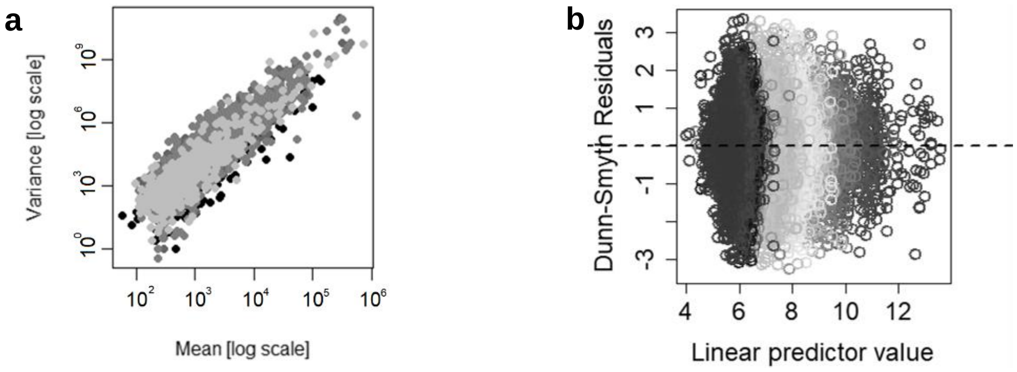

Supplementary Figure 5 Check plots for general linear multivariate models (GLMMs) regression using a negative-binomial distribution on the metagenomics microbial community dataset (top-500 genera) from day 35 of the study. Each point represents an OTU, while tones of grey represent different disturbance levels. (**a**) Mean-variance plot showing that the mean increases with the variance, justifying the use of a negative-binomial distribution. (**b**) Residual vs. fitted plot to check the goodness of fit for the negative binomial model. No clear pattern suggests the assumption is acceptable. Analysis of deviance for such GLMMs was significant, including multiple comparisons correction ($P^{BH} = 0.0149$).

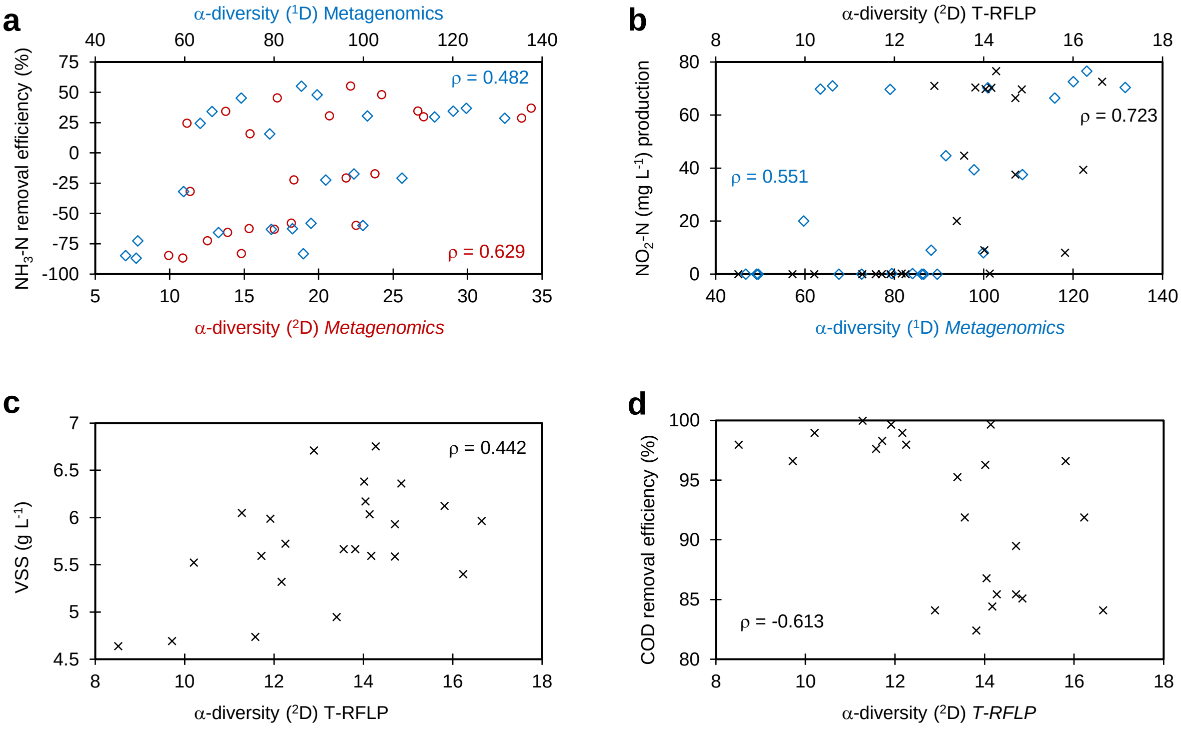

Supplementary Figure 6 Spearman's correlations between α-diversity and ecosystem function. Robust estimators of α-diversity indexes ^{1}D and ^{2}D were employed. Variation of (**a**) NH_3–N removal efficiency (negative values represent accumulation), (**b**) NO_2–N production, (**c**) VSS (biomass), and (**d**) COD (organic carbon) removal efficiency as a function of α-diversity indexes from Metagenomics (^{1}D, ^{2}D, bacterial genus taxonomy level) and T-RFLP (^{2}D) datasets. Only significant correlations after 10% FDR correction are reported. Correlation coefficients (ρ) are included, using the same font color as the x-axis and data points they are referred to. Data from day 35 of the study.

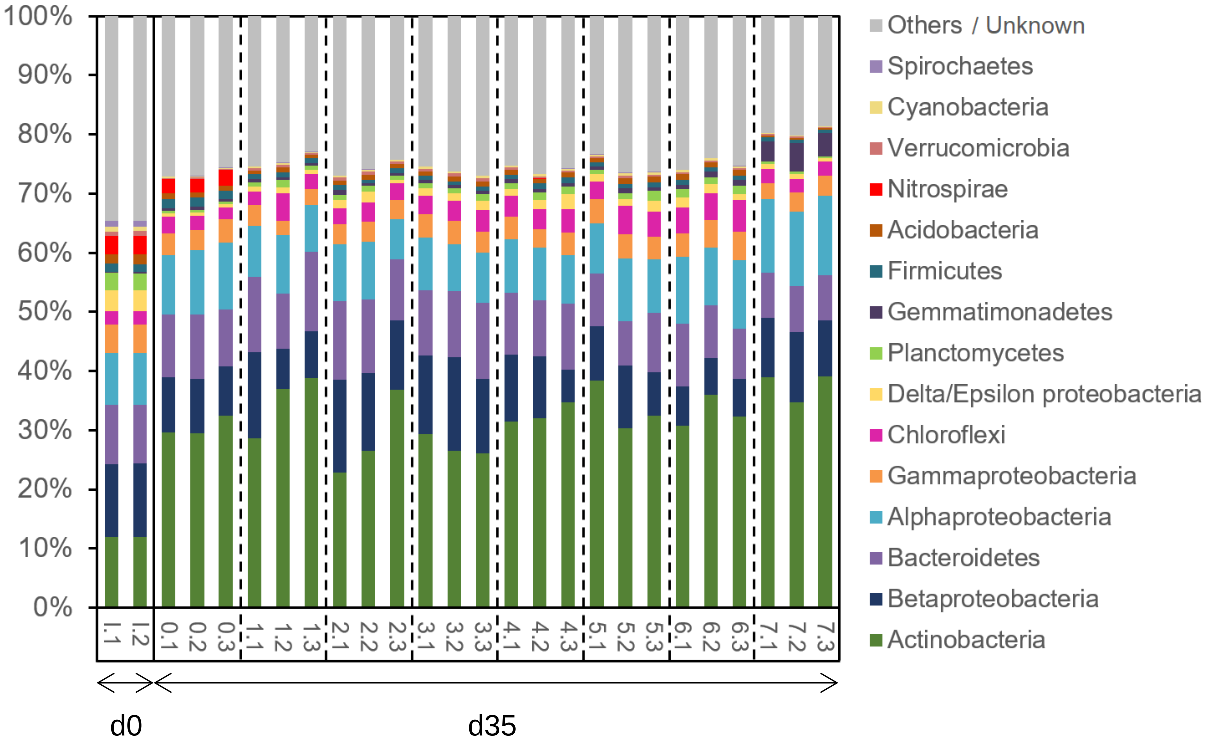

Supplementary Figure 7 Relative abundances of major bacterial phyla as assessed by metagenomics at the beginning (d0) and end (d35) of the study. Each columns represents a replicate. First character below columns indicates: I, WWTP inoculum; 0-7, disturbance levels. Second character below columns represents independent replicate number.

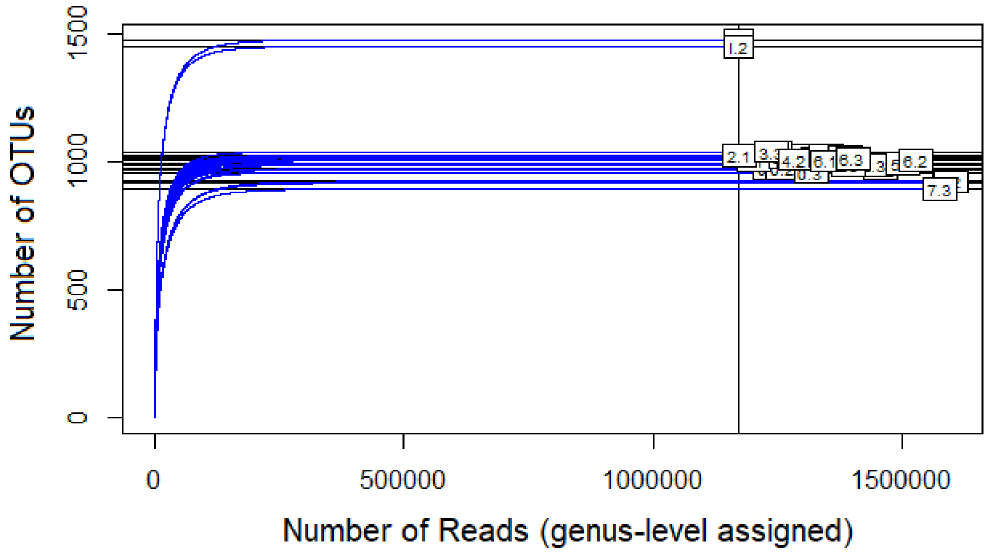

Supplementary Figure 8 Rarefaction curves for the metagenomics dataset samples (reads assigned to genus taxonomic level) corresponding to d0 (n = 2) and d35 (n = 24). Each blue curve represents a sample. First character on each box indicates: I, WWTP inoculum; 0-7, disturbance levels. Second character below columns represents independent replicate number.

Supplementary Tables

Supplementary Table 1 Multivariate tests for relative abundances of communities employing disturbance and time as factors.

Source** (factor§)	Distance Metric‡‡	n¶	PERMANOVA†			PERMDISP‡			
			**df#**	**pseudo-F**	**$P^{BH\|\|}$**	**df1**	**df2**	**F**	**$P^{BH\|\|}$**
All (L)	BC	96	7	15.13◊	**0.0003**	7	88	1.34	0.3521
All (T)	BC	96	3	24.49◊	**0.0003**	3	92	6.57	**0.0036**
14 d (L)	BC	24	7	2.21	0.1505	7	16	3.04	0.1505
21 d (L)	BC	24	7	6.02	**0.0003**	7	16	2.92	0.3521
28 d (L)	BC	24	7	5.19	**0.0003**	7	16	5.53	**0.0432**
35 d (L)	BC	24	7	5.81	**0.0003**	7	16	4.39	0.1505
35 d (L)††	BC	24	7	9.09	**0.0003**	7	16	9.79	**0.0141**

† Permutational multivariate analysis of variance (semi-parametric)

‡ Test for homogeneity of multivariate dispersions (semi-parametric)

§ L: disturbance levels, T: time

¶ Number of samples involved in the test

|| P-values calculated using 9,999 permutations and corrected for multiple comparisons with a Benjamini-Hochberg's FDR of 10% (significant ones in bold)

Degrees of freedom

◊ Factor interaction for all data points (LxT) was not significant (pseudo-F = 2.21).

** Time point analyzed ('all' means the whole dataset)

†† Data from metagenomics dataset at the bacterial Genus taxonomy level (T-RFLP data otherwise)

‡‡ Dissimilarity metric employed for the multivariate tests. BC: Bray-Curtis.

Supplementary Table 2 Mantel and Procrustes tests comparing Bray-Curtis distance matrices from metagenomics (genus level) and T-RFLP microbial community datasets on day 35 of the study.

Ordination		**Mantel**		**PROTEST**[†]		
**Method**[*]	**n**[‡]	**r**[§]	$\mathbf{P^{BH}}$	$\mathbf{m^2}$[¶]	**R**[‖]	$\mathbf{P^{BH}}$
PCO	24	0.730	**0.002**	0.514	0.697	**<0.002**
NMDS				0.580	0.648	**<0.002**

[*] Tests were performed on square-root transformed Bray-Curtis dissimilarity matrixes

[†] Procrustes Test

[‡] Number of samples involved in the test

[§] Pearson product-moment correlation coefficient

[¶] Sum of squares statistic for PROTEST

[‖] PROTEST correlation in Procrustes rotation

Supplementary References

1 Hesselmann, R. P. X., Werlen, C., Hahn, D., van der Meer, J. R. & Zehnder, A. J. B. Enrichment, phylogenetic analysis and detection of a bacterium that performs enhanced biological phosphate removal in activated sludge. *Syst. Appl. Microbiol.* **22**, 454-465 (1999).

2 Falk, M. W. & Wuertz, S. Effects of the toxin 3-chloroaniline at low concentrations on microbial community dynamics and membrane bioreactor performance. *Water Res.* **44**, 5109-5115 (2010).

3 APHA-AWWA-WEF. *Standard methods for the examination of water and wastewater*. 22 edn, (APHA-AWWA-WEF, 2005).

4 Albertsen, M., Hansen, L. B. S., Saunders, A. M., Nielsen, P. H. & Nielsen, K. L. A metagenome of a full-scale microbial community carrying out enhanced biological phosphorus removal. *ISME J.* **6**, 1094-1106 (2012).

5 Falk, M. W., Song, K. G., Matiasek, M. G. & Wuertz, S. Microbial community dynamics in replicate membrane bioreactors - Natural reproducible fluctuations. *Water Res.* **43**, 842-852 (2009).

6 Seshan, H. *The effects of perturbations on microbial community dynamics and process performance in wastewater bioreactors* PhD thesis, University of California, Davis, (2015).

7 Dumbrell, A. J., Nelson, M., Helgason, T., Dytham, C. & Fitter, A. H. Relative roles of niche and neutral processes in structuring a soil microbial community. *ISME J.* **4**, 337-345 (2010).

8 Ayarza, J. M. & Erijman, L. Balance of neutral and deterministic components in the dynamics of activated sludge floc assembly. *Microb. Ecol.* **61**, 486-495 (2011).

9 Shade, A. *et al.* Lake microbial communities are resilient after a whole-ecosystem disturbance. *ISME J.* **6**, 2153-2167 (2012).

10 Falk, M. W., Seshan, H., Dosoretz, C. & Wuertz, S. Partial bioaugmentation to remove 3-chloroaniline slows bacterial species turnover rate in bioreactors. *Water Res.* **47**, 7109-7119 (2013).

11 Pholchan, M. K., Baptista, J. D., Davenport, R. J., Sloan, W. T. & Curtis, T. P. Microbial community assembly, theory and rare functions. *Front. Microbiol.* **4**, 1-9 (2013).

12 Winter, C., Matthews, B. & Suttle, C. A. Effects of environmental variation and spatial distance on Bacteria, Archaea and viruses in sub-polar and arctic waters. *ISME J.* **7**, 1507-1518 (2013).

13 Kim, D. Y. *et al.* Monthly to interannual variability of microbial eukaryote assemblages at four depths in the eastern North Pacific. *ISME J.* **8**, 515-530 (2014).

14 Lear, G., Bellamy, J., Case, B. S., Lee, J. E. & Buckley, H. L. Fine-scale spatial patterns in bacterial community composition and function within freshwater ponds. *ISME J.* **8**, 1715-1726 (2014).

15 Campbell, A. H., Marzinelli, E. M., Gelber, J. & Steinberg, P. D. Spatial variability of microbial assemblages associated with a dominant habitat-forming seaweed. *Front. Microbiol.* **6** (2015).

16 Ofiteru, I. D. *et al.* Combined niche and neutral effects in a microbial wastewater treatment community. *Proc. Natl. Acad. Sci. USA* **107**, 15345-15350 (2010).

17 Powell, J. R. *et al.* Deterministic processes vary during community assembly for ecologically dissimilar taxa. *Nat. Commun.* **6**, 1-10 (2015).

18 Bent, S. J., Pierson, J. D. & Forney, L. J. Measuring species richness based on microbial community fingerprints: The emperor has no clothes. *Appl. Environ. Microbiol.* **73**, 2399-2399 (2007).
19 Bent, S. J. & Forney, L. J. The tragedy of the uncommon: understanding limitations in the analysis of microbial diversity. *ISME J.* **2**, 689-695 (2008).
20 van Dorst, J. *et al.* Community fingerprinting in a sequencing world. *FEMS Microbiol. Ecol.* **89**, 316-330 (2014).
21 Dowd, S. E. *et al.* Evaluation of the bacterial diversity in the feces of cattle using 16S rDNA bacterial tag-encoded FLX amplicon pyrosequencing (bTEFAP). *BMC Microbiol.* **8** (2008).
22 Degnan, P. H. & Ochman, H. Illumina-based analysis of microbial community diversity. *ISME J.* **6**, 183-194 (2012).
23 Singh, B. K. & Thomas, N. Multiplex-terminal restriction fragment length polymorphism. *Nat. Protoc.* **1**, 2428-2433 (2006).
24 Kaplan, C. W. & Kitts, C. L. Bacterial succession in a petroleum land treatment unit. *Appl. Environ. Microbiol.* **70**, 1777-1786 (2004).
25 Abdo, Z. *et al.* Statistical methods for characterizing diversity of microbial communities by analysis of terminal restriction fragment length polymorphisms of 16S rRNA genes. *Environ. Microbiol.* **8**, 929-938 (2006).
26 Anderson, M. J. & Walsh, D. C. I. PERMANOVA, ANOSIM, and the Mantel test in the face of heterogeneous dispersions: What null hypothesis are you testing? *Ecol. Monogr.* **83**, 557-574 (2013).
27 Pagaling, E. *et al.* Community history affects the predictability of microbial ecosystem development. *ISME J.* **8**, 19-30 (2014).
28 Clarke, K. R. & Gorley, R. N. *PRIMER v7: User Manual/Tutorial*. (PRIMER-E, 2015).
29 Anderson, M. J. & Willis, T. J. Canonical analysis of principal coordinates: A useful method of constrained ordination for ecology. *Ecology* **84**, 511-525 (2003).
30 Clarke, K. R. Nonparametric multivariate analyses of changes in community structure. *Aust. J. Ecol.* **18**, 117-143 (1993).
31 Legendre, P. & Legendre, L. *Numerical ecology*. 3 edn, (Elsevier, 2012).
32 Anderson, M. J. Distance-based tests for homogeneity of multivariate dispersions. *Biometrics* **62**, 245-253 (2006).
33 Hill, M. O. Diversity and evenness: a unifiying notation and its consequences. *Ecology* **54**, 427-432 (1973).
34 Vuono, D. C. *et al.* Disturbance and temporal partitioning of the activated sludge metacommunity. *ISME J.* **9**, 425-435 (2015).
35 Haegeman, B. *et al.* Robust estimation of microbial diversity in theory and in practice. *ISME J.* **7**, 1092-1101 (2013).
36 Peter, H. *et al.* Function-specific response to depletion of microbial diversity. *ISME J.* **5**, 351-361 (2011).
37 Benjamini, Y. & Hochberg, Y. Controlling the false discovery rate: a practical and powerful approach to multiple testing. *Journal of the Royal Statistical Society. Series B (Methodological)* **57**, 289-300 (1995).
38 Kraft, N. J. B. *et al.* Disentangling the Drivers of β Diversity Along Latitudinal and Elevational Gradients. *Science* **333**, 1755-1758 (2011).
39 Zhou, J. Z. et al. Stochasticity, succession, and environmental perturbations in a fluidic ecosystem. Proc. Natl. Acad. Sci. USA 111, E836-E845 (2014).
